# Antibodies to carbamylated α-enolase epitopes in rheumatoid arthritis also bind citrullinated epitopes and are largely indistinct from anti-citrullinated protein antibodies

**DOI:** 10.1186/s13075-016-1001-6

**Published:** 2016-05-04

**Authors:** Evan Reed, Xia Jiang, Nastya Kharlamova, A. Jimmy Ytterberg, Anca I. Catrina, Lena Israelsson, Linda Mathsson-Alm, Monika Hansson, Lars Alfredsson, Johan Rönnelid, Karin Lundberg

**Affiliations:** Rheumatology Unit, Department of Medicine, Karolinska Institutet, Stockholm, Sweden; Cardiovascular Epidemiology, Institute of Environmental Medicine, Karolinska Institutet, Stockholm, Sweden; Division of Physiological Chemistry, Department of Medical Biochemistry and Biophysics, Karolinska Institutet, Stockholm, Sweden; ThermoFisher Scientific, Uppsala, Sweden; Centre for Occupational and Environmental Medicine, Stockholm County Council, Stockholm, Sweden; Department of Immunology, Genetics and Pathology, Uppsala University, Uppsala, Sweden

**Keywords:** Anti-citrullinated protein antibodies, Anti-carbamylated protein antibodies, Rheumatoid arthritis, Cross-reactivity

## Abstract

**Background:**

In addition to anti-citrullinated protein antibodies (ACPAs), antibodies targeting carbamylated (i.e., homocitrullinated) proteins (anti-CarP antibodies) have been described in rheumatoid arthritis (RA). However, the extent to which anti-CarP antibodies are truly distinct from ACPA remains unclear, and few studies have focused on specific autoantigens. Here, we examine cross-reactivity between ACPA and anti-CarP antibodies, in the context of the candidate autoantigen α-enolase.

**Methods:**

Cross-reactivity was examined by immunoblotting of citrullinated and carbamylated proteins using purified ACPA; and by peptide absorption experiments, using the citrullinated α-enolase peptide CEP-1 and a homocitrulline-containing version (carb-CEP-1) in ELISA. The population-based case-control cohort EIRA (n = 2836 RA; 373 controls) was screened for reactivity with CEP-1 and carb-CEP-1, using the ISAC multiplex array. Associations between anti-CarP antibodies, smoking and genetic risk factors were analysed using unconditional logistic regression models. Differences in antibody levels were investigated using the Mann-Whitney *U* test.

**Results:**

Affinity-purified ACPA was found to bind carbamylated proteins and homocitrulline-containing peptides, demonstrating definitive cross-reactivity between ACPA and anti-CarP antibodies. Anti-carb-CEP-1 reactivity in EIRA was almost exclusively confined to the CEP-1-positive subset, and this group of RA patients (21 %) displayed a particularly strong ACPA response with marked epitope spreading. The small RA subset (3 %) with homocitrulline reactivity in the absence of citrulline reactivity did not associate with smoking or risk genes, and importantly had significantly lower anti-carb-CEP-1 antibody levels.

**Conclusion:**

Our data presented herein cast doubt on the specificity of anti-CarP antibodies in RA, which we posit may be a subset of cross-reactive ACPA.

**Electronic supplementary material:**

The online version of this article (doi:10.1186/s13075-016-1001-6) contains supplementary material, which is available to authorized users.

## Background

Autoantibodies to citrullinated proteins (ACPA) are today a well-known and accepted feature of rheumatoid arthritis (RA) [1, 2]. These autoantibodies have been linked to RA risk factors, most notably *HLA-DRB1* shared epitope (SE) alleles and cigarette smoking, and their presence predicts a more destructive disease process [3–7]. However, despite the identification of several putative citrullinated autoantigens, including fibrinogen [8], vimentin [9], type II collagen [10], α-enolase [11] and histone 4 [12], the specific in vivo ACPA targets triggering autoimmunity and driving disease remain obscure.

More recently, antibodies to carbamylated proteins containing homocitrulline (anti-CarP antibodies) were described in RA [13]. Protein carbamylation, or homocitrullination, is an enzyme-independent post-translational modification of lysine residues by isocyanate, present in, for example, cigarette smoke [14]. As smoking is a well-described risk factor for RA [15, 16], it has been proposed that smoking could be linked to anti-CarP antibodies in RA via increased carbamylation and the subsequent production of anti-CarP antibodies [17–19]. However, scientific data in support of this hypothesis has yet to be presented. Anti-CarP antibodies are specific for RA [20] and reportedly distinct from ACPA, based on the detection of anti-CarP antibodies in ACPA-negative disease [13, 21, 22]. However, in the Swedish Epidemiological Investigation of RA (EIRA) study and in the Dutch Early Arthritis Clinic (EAC) cohort, we recently showed that only 4–7 % of RA patients were anti-CarP antibody-positive in the absence of ACPA. Notably, there was no specific association between *HLA-DRB1* SE or smoking and anti-CarP antibodies, when the analyses were adjusted for the presence of ACPA [21].

In addition, the widely used biochemical assay for detection of peptidylcitrulline, the so-called Senshu method [23] where rabbit polyclonal antibodies bind chemically modified citrulline residues, was found to also detect homocitrulline [24] and purified ACPA have been shown to bind not only citrullinated fibrinogen, but also carbamylated fibrinogen [20].

The extent to which these two autoantibody specificities are cross-reactive, and the association between these antibodies and environmental and genetic risk factors for RA, has not been thoroughly explored, and as yet, only fibrinogen and more recently vimentin have been studied in this context [13, 20, 21, 24–26]. Therefore, it is imperative that more work on specific antigens is performed in order to fully understand the relationship between ACPA and anti-CarP antibodies in the aetiopathology of RA [19].

Citrullinated α-enolase has long been scrutinized as a potential target for ACPA in RA [11, 27–34]. Antibodies to CEP-1, the immunodominant B cell epitope of citrullinated α-enolase [27] are found in approximately 40 % of patients with RA, and have been associated with SE, *PTPN22* and smoking [28, 32]. Hence, in the present study, we have investigated the antibody responses to citrullinated and carbamylated α-enolase and their relation to RA risk factors in the Swedish population-based case-control cohort EIRA.

## Methods

### Patients

The present study includes patients newly diagnosed with RA (cases) and age-matched, sex-matched and residential area-matched controls from the Swedish Epidemiological Investigation of RA (EIRA) cohort. Information on cigarette smoking (“ever smoker” or “never smoker”) was obtained via self-reported questionnaire at baseline [16]. Genotyping of *HLA-DRB1* shared epitope (SE) alleles and the protein tyrosine phosphatase gene (*PTPN22* rs2476601) was performed on blood samples obtained within one week of the RA diagnosis [5, 35]. Smoking and genetic data for the present study were retrieved from the EIRA database on 2784, 2235 and 2477 patients with RA and 4864, 1923 and 1936 controls, for smoking, SE and *PTPN22,* respectively. For antibody purification, plasma samples from patients with RA with a strong anti-CEP-1 antibody response (n = 5) or a strong anti-CCP2 antibody response (n = 38) were collected at the Rheumatology Clinic, Karolinska University Hospital Solna, Stockholm, Sweden. Informed consent was obtained from participating patients and controls, and ethical approvals for the study was granted at the regional ethics review board at Karolinska Institutet, Stockholm, Sweden.

### Antigens

Three cyclic peptides corresponding to amino acid 5-21 of full-length α-enolase were synthesized by Innovagen (Malmö, Sweden): the original CEP-1 peptide containing two citrulline residues (CEP-1) [27]; the arginine-containing control peptide REP-1; and a version of CEP-1 containing homocitrulline in the place of citrulline, denoted carb-CEP-1 (Additional file 1: Table S1).

Recombinant human α-enolase, produced in-house, and purified human fibrinogen (Enzyme Research, South Bend, IN, USA) depleted of immunoglobulins, were citrullinated or carbamylated in vitro. Citrullination was performed for 2 h at 37 °C, at a protein concentration of 1 mg/ml, in peptidylarginine deiminase (PAD) buffer (100 mM Tris, 10 mM CaCl2, 5 mM dithiothreitol (DTT), pH 7.6) using 2 U/mg of rabbit skeletal muscle PAD2 enzyme (Sigma, St. Louis, MO, USA). The reaction was stopped by the addition of 20 mM ethylenediaminetetraacetic acid (EDTA), followed by extensive dialysis to calcium-free PBS. Carbamylated proteins were produced by incubating α-enolase and fibrinogen in PBS at 1 mg/ml in the presence of 100 mM potassium cyanate (KOCN) (Sigma) overnight at 37 °C, followed by extensive dialysis to calcium-free PBS. Successful citrullination and carbamylation were confirmed by mass spectrometry (data not shown). For a detailed description of the mass spectrometry analysis see Additional file 1: Supplementary methods.

### Affinity purification of ACPA IgG

Plasma samples (n = 43) were centrifuged and diluted in PBS (1:5 v/v) before applied to Protein G HP columns (GE Healthcare) for whole IgG enrichment. To further purify CEP-1-specific IgG, REP-1 and CEP-1 peptides (1 mg/ml) were directly coupled to 1 ml NHS-Sepharose columns (GE Healthcare), and anti-CEP-1 IgG from five anti-CEP-1 antibody-positive serum samples was subsequently purified from whole IgG using the CEP-1 affinity column, after pre-absorption on the REP-1 column to remove non-citrulline-specific antibodies. Bound antibodies were eluted with 0.1 M glycine-HCl (pH 2.7) and directly neutralized with 1 M Tris (pH 9). Column flow-through (FT) fractions depleted of anti-CEP-1 IgG were collected in parallel. Microsep^TM^ UF Centrifugal Devices (Pall Life Science, Port Washington, NY, USA) were used in accordance with the manufacturer’s instructions, to concentrate the antibodies and to change the buffer into PBS. Anti-CCP2-reactive IgG from 38 anti-CCP2-positive RA serum samples were pooled after purification on CCP2-columns kindly donated by EuroDiagnostica AB, Malmö, Sweden, as previously described [36].

### Antibody detection using ISAC and ELISA

High-throughput anti-CEP-1, anti-REP-1 and anti-carb-CEP-1 antibody screening of serum samples from 2836 patients with RA from the EIRA cohort and 373 EIRA controls was accomplished using a custom-made microarray based on the ImmunoCAP immuno solid-phase allergen chip multiplex assay (ISAC) microarray system (Phadia AB, Uppsala, Sweden) containing the peptides of interest, as previously described [25, 37]. This microarray also contains a large number of other citrullinated peptides derived from different proteins, including fibrinogen, vimentin and collagen type II, and their corresponding arginine-containing control peptides. Cut offs for antibody positivity were calculated based on the 98th percentile among the EIRA controls. A detailed description of the ISAC method is provided in Additional file 1.

For testing the reactivity of the affinity-purified anti-CEP-1 and FT IgG fractions, and for analysing the degree of cross-reactivity between double-positive (CEP-1^+^/Carb-CEP-1^+^) or single-positive (CEP-1^+^/Carb-CEP-1^-^ and CEP-1^-^/Carb-CEP-1^+^) EIRA RA serum samples, peptide ELISAs detecting anti-CEP-1 and anti-Carb-CEP-1 IgG were used as previously described [27, 28, 32] (see Additional file 1: Supplementary methods for details).

### Cross-reactivity assay

Anti-CEP-1/anti-Carb-CEP-1 double-positive serum samples (n = 4), anti-CEP-1 single-positive (n = 4), and anti-Carb-CEP-1 single-positive serum samples (n = 4) were selected for the cross-reactivity experiment. Serum samples were diluted 1:100 in radioimmunoassay (RIA) buffer and incubated with 100 μg/ml of the CEP-1 or the Carb-CEP-1 peptide for 2 h at room temperature (RT). Following incubation, the absorbed serum was analysed using the same protocol as for the peptide ELISA described previously (see also Additional file 1).

### Western blot

Citrullinated, carbamylated and unmodified proteins (100 ng/well) were separated on NuPAGE® Bis-Tris 4-20 % gels (Bio-Rad Laboratories, Hercules, CA, USA) and transferred to nitrocellulose membranes. Membranes were blocked with 5 % milk in Tris-buffered saline/0.05 % Tween and probed with a pool (n = 38) of purified anti-CCP2 IgG (or the corresponding CCP2 column FT IgG pool) at 2 μg/ml overnight at 4 °C, then washed with PBS/0.05 % Tween and incubated with HRP-conjugated goat anti-human IgG (Jackson ImmunoResearch, West Grove, PA, USA), diluted 1:10,000, for 1 h at RT. Bound antibody was detected using ECL chemiluminescence (GE Healthcare).

### Statistics

Patients were divided into four different subsets according to the presence or absence of anti-CEP-1 and anti-carb-CEP-1 IgG. The odds ratio (OR) and 95 % confidence intervals (CI) for each RA subset, in relation to smoking, SE and *PTPN22*, were calculated separately through unconditional logistic regression models, adjusted for matching variables (age, gender and residential area). Exposed individuals were compared with unexposed individuals (smokers vs. non-smokers, carriers of any copy of SE vs. non-carriers, carriers of the *PTPN22* risk allele vs*.* non-carriers). All analyses were implemented through SAS V.9.3. Statistical differences in antibody levels and number of ACPA fine specificities, between different subsets, were determined by the Mann-Whitney *U* test for independent groups. The same method was also used to determine the relationship between anti-carb-CEP-1 antibody levels and SE/smoking.

## Results

### Purified ACPA IgG bind carbamylated proteins

**Fig. 1:**
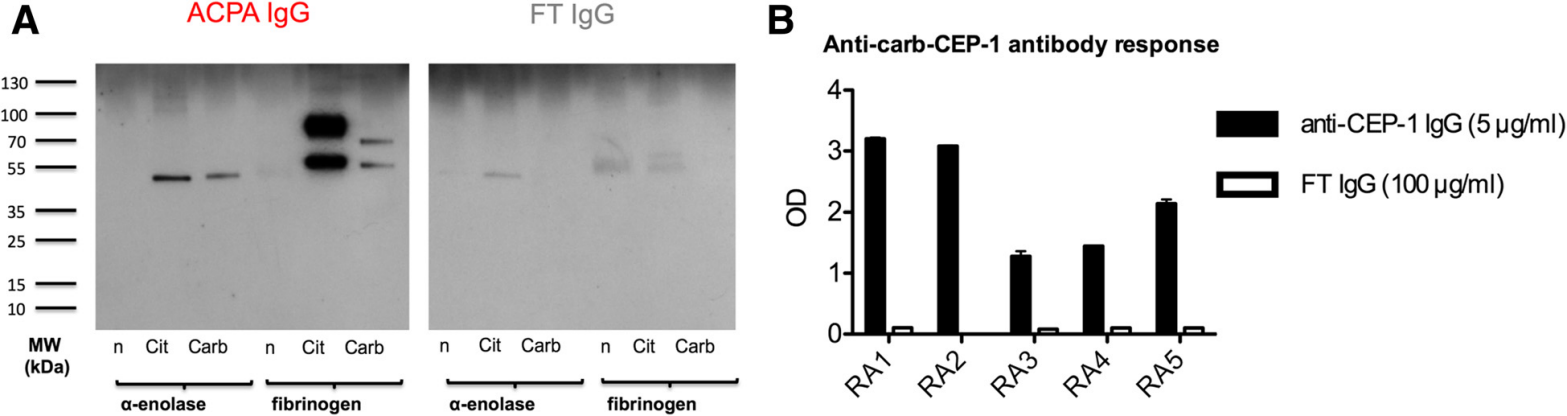
Human anti-citrullinated protein antibodies (*ACPA*) cross-react with carbamylated epitopes. **a** Native (*n*), in vitro citrullinated (*Cit*) and in vitro carbamylated (*Carb*) samples of recombinant human α-enolase and purified human fibrinogen were subjected to western blot using affinity-purified anti-CCP2 IgG (ACPA IgG) and CCP2-depleted column flow-through IgG (*FT IgG*), obtained from a pool of 38 rheumatoid arthritis (*RA*) serum samples. **b** Affinity-purified anti-CEP-1 IgG and the corresponding column FT IgG fractions from five patients with RA were tested for reactivity to the carb-CEP-1 peptide in ELISA; antibody levels are presented as optical density (*OD*)

Using a pool of affinity-purified anti-CCP2 IgG, previously described to bind both citrullinated α-enolase and fibrinogen [36], we could demonstrate cross-reactivity of human ACPA with carbamylated α-enolase for the first time, and in line with previous reports [20, 24], we could also show cross-reactivity with carbamylated fibrinogen (Fig. 1a). There was no reactivity against unmodified proteins. The corresponding FT IgG pool bound neither modified nor native proteins; only some weak unspecific background staining was observed.

### Purified anti-CEP-1 IgG displays cross-reactivity with a homocitulline-containing version of CEP-1

To further investigate the specificity and extent of cross-reactivity between citrullinated and carbamylated epitopes, we focused on α-enolase and the immunodominant CEP-1 epitope. Affinity-purified anti-CEP-1-specific IgG bound not only CEP-1 in ELISA, but also a version of CEP-1 (denoted carb-CEP-1) identical in sequence but with citrulline residues replaced with homocitrullines. Anti-CEP-1 IgG purified from different patients with RA showed consistently strong binding to the CEP-1 peptide in ELISA (data not shown), and in addition displayed varying degrees of binding to the carb-CEP-1 peptide (Fig. 1b). Flow-through IgG from the same five patients did not bind to CEP-1 or carb-CEP-1, demonstrating that the carb-CEP-1 reactivity was confined to the anti-CEP-1 IgG eluate fraction. None of the anti-CEP-1 IgG column eluates demonstrated reactivity to the control peptide REP-1 (data not shown). Taken together, these data suggest that citrullinated α-enolase-specific ACPA also have the ability to bind homocitrulline-containing epitopes.

### Anti-carb-CEP-1 reactivity in relation to anti-CEP-1 status in EIRA

**Fig. 2:**
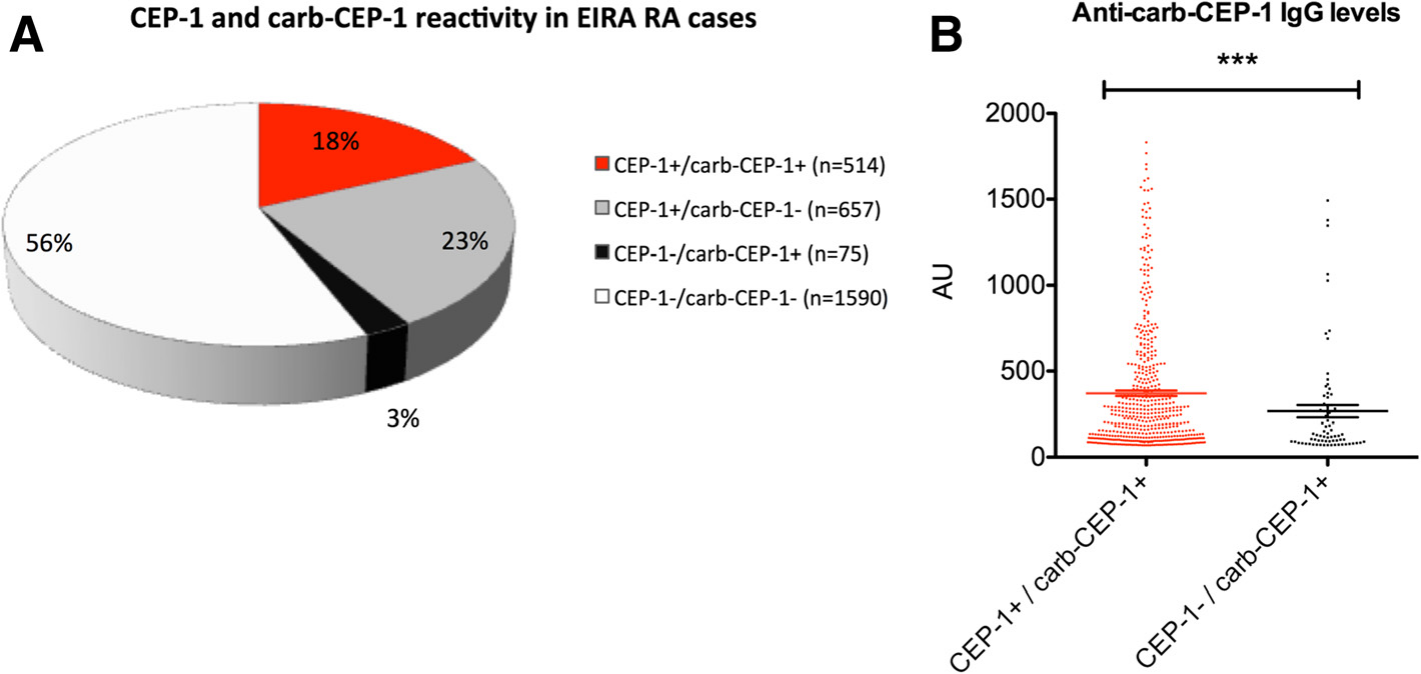
Reactivity to CEP-1 and homocitrullinated CEP-1 peptide (*carb-CEP-1*) in Swedish Epidemiological Investigation of Rheumatoid Arthritis (*EIRA*) rheumatoid arthritis (*RA*) cases. **a** Sera from 2836 patients with RA from the EIRA cohort were tested for reactivity with CEP-1 and carb-CEP-1 using the immuno solid-phase allergen chip multiplex assay (ISAC) platform and divided into subsets. **b** Anti-carb-CEP-1 antibody levels were compared between the CEP-1^+^/carb-CEP-1^+^ and CEP-1^-^/carb-CEP-1^+^ subsets. Antibody levels are presented as arbitrary units (*AU*)

Using the large EIRA case-control cohort, we next sought to determine the proportion of patients with RA with antibodies binding to carb-CEP-1, and how this reactivity correlated with CEP-1 positivity. Reactivity to CEP-1, REP-1 and carb-CEP-1 was therefore analysed in serum from 2836 RA cases using the ISAC platform. The frequency of anti-CEP-1 antibody-positive patients with RA was 41 %, which is in accordance with previous analyses using a smaller proportion of EIRA (n = 1985 RA cases) and the ELISA method [28, 32]. There was less than 2 % reactivity towards the REP-1 control peptide, while 21 % of patients with RA had reactivity towards the carb-CEP-1 peptide (Fig. 2a). Notably, almost all patients positive for anti-carb-CEP-1 IgG were also positive for antibodies to CEP-1. Only 3 % of EIRA RA serum samples had a unique and specific reactivity with carb-CEP-1. Importantly, anti-carb-CEP-1 antibody levels were significantly lower in patients who were carb-CEP-1 single-positive than in the CEP-1/carb-CEP-1 double-positive RA subset (Fig. 2b). Furthermore, when increasing specificity to 100 %, the carb-CEP-1 single-positive subset was almost completely eliminated (<1 %) while the double-positive subset remained (data not shown), suggesting that carb-CEP-1 reactivity in the absence of CEP-1 reactivity is extremely rare.

### Cross-reactivity between CEP-1 and carb-CEP-1

**Fig. 3:**
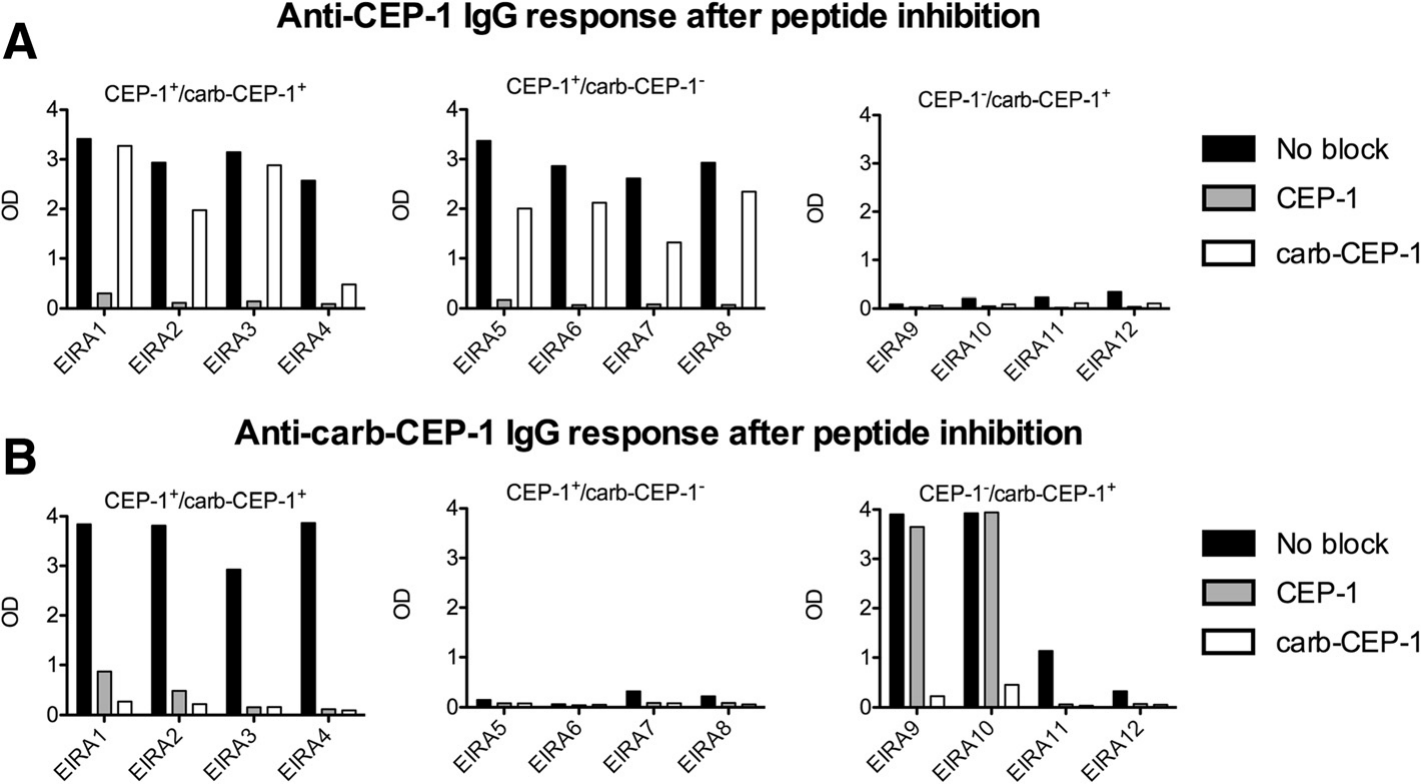
Homocitrullinated CEP-1 peptide (*Carb-CEP-1*) reactivity results from cross-reactive anti-CEP-1 antibodies. To assess cross-reactivity between CEP-1 and carb-CEP-1, serum from patients positive for either anti-CEP-1 antibodies only (n = 4) or for anti-carb-CEP-1 antibodies only (n = 4), and serum from patients positive for both anti-CEP-1 and anti-carb-CEP-1 antibodies (n = 4), were pre-absorbed by incubating with either dilution buffer, CEP-1 peptide or carb-CEP-1 peptide. Levels of anti-CEP-1 (**a**) and anti-carb-CEP-1 (**b**) antibodies were subsequently measured by ELISA, and presented as optical density (*OD*). *EIRA* Swedish Epidemiological Investigation of Rheumatoid Arthritis cohort

In order to more directly determine whether the anti-carb-CEP-1 antibody response was simply the result of homocitrulline-cross-reactive anti-CEP-1 antibodies, we subsequently performed peptide absorption experiments. In these experiments we demonstrated that ACPA from different patients with RA displayed varying degrees of cross-reactivity to the homocitrulline-containing CEP-1 homologue, carb-CEP-1. In a selection of 16 RA serum samples, we examined whether the CEP-1 peptide and/or the carb-CEP-1 peptide could inhibit antibody binding to CEP-1 and/or carb-CEP. Not surprisingly, in CEP-1 positive sera, pre-incubation with the CEP-1 peptide completely inhibited (100 %) all CEP-1 reactivity, while inhibition with the carb-CEP-1 peptide was less efficient (4–49 %) in blocking binding to CEP-1 (Fig. 3a). Notably, the anti-carb-CEP-1 antibody response in double-positive sera was blocked efficiently by both CEP-1 and carb-CEP-1 pre-incubation, while in the rare group of carb-CEP-1 single-positive subjects, more varying degrees of inhibition was seen (Fig. 3b). Two serum samples had almost complete inhibition of binding to carb-CEP-1 after both CEP-1 and carb-CEP-1 pre-incubation, while the CEP-1 peptide did not inhibit binding to carb-CEP-1 in the two serum samples that displayed a very strong anti-carb-CEP-1 IgG response (optical density (OD) values >3.5 AU/ml).

### Anti-carb-CEP-1 reactivity in relation to smoking in EIRA

Based on the link between smoking and carbamylation and the association between smoking and RA [14–16], we next investigated the role of smoking in the development of anti-carb-CEP-1 antibodies. Our first analysis showed that smoking was associated with anti-carb-CEP-1 positivity, and with elevated anti-carb-CEP-1 antibody levels (data not shown). However, we have previously shown that smoking is strongly associated with anti-CEP-1 antibodies [28, 32], and as the majority of anti-carb-CEP-1 reactivity was confined to the CEP-1-positive subset, we also had to consider CEP-1 reactivity in the analysis. Hence, we divided the patients with RA into four subsets, based on presence or absence of carb-CEP-1 and/or CEP-1 reactivity. With this division, we found that smoking was significantly associated with anti-CEP-1 single-positive RA, with an odds ratio of 2.21 (95 % CI 1.82, 2.68), and with CEP-1/carb-CEP-1 double-positive disease (OR = 2.6; 95 % CI 2.07, 3.25), but not with anti-carb-CEP-1 single-positive disease (OR = 1.15; 95 % CI 0.71, 1.86) (Table 1). Importantly, there was no statistical difference when comparing ORs for the double-positive vs. the CEP-1 single-positive subset (2.6 vs*.* 2.21, *p* = 0.37), suggesting that smoking has no specific effect on the development of anti-CarP antibodies, in line with our previous data [21].

**Table 1:** Association between smoking and RA in subgroups of patients, divided based on the presence/absence of anti-CEP-1 and anti-carb-CEP-1 antibodies

CEP-1/carb-CEP-1	Smoking		OR^a^ (95 % CI)
	Never	Ever	
Controls	2114 (43.46)	2750 (56.54)	Reference 1.0
–/–	575 (37.00)	979 (63.00)	1.23^b^ (1.09, 1.39)
+/–	159 (24.54)	489 (75.46)	2.21^b^ (1.82, 2.68)
–/+	29 (38.67)	46 (61.33)	1.15 (0.71, 1.86)
+/+	107 (21.10)	400 (78.90)	2.60^b^ (2.07, 3.25)

*CEP-1* citrullinated α-enolase peptide-1, *carb-CEP-1* carbamylated CEP-1, *CI* confidence interval, *RA* rheumatoid arthritis. ^a^Odds ratios (*OR*) were adjusted for age, gender and residential area. ^b^Statistically significant ORs

### Anti-carb-CEP-1 reactivity in relation to *HLA-DRB1* SE in EIRA

A similar analysis to that used for smoking was also performed for *HLA-DRB1* SE, and again there was significant association between *HLA-DRB1* SE and both anti-carb-CEP-1 antibody positivity and elevated anti-carb-CEP-1 antibody levels (data not shown). When subdividing patients, the association with the SE was significantly stronger in CEP-1 single-positive RA (OR = 6.58; 95 % CI 5.01, 8.65) than with carb-CEP-1 single-positive RA (OR = 2.07; 95 % CI 1.19, 3.61), (6.58 vs*.* 2.07, *p* = 0.0002) (Table 2). However, contrary to the smoking data, the association between the SE and the double-positive subset (OR = 10.42; 95 % CI 7.2, -14.90) was significantly stronger than with the CEP-1 single-positive subset (10.42 vs. 6.58, *p* = 0.03), suggesting an SE-mediated effect on the development of carb-(cross)-reactive antibodies.

**Table 2:** Association between *HLA-DRB1* SE alleles and RA in subgroups of patients, divided based on the presence/absence of anti-CEP-1 and anti-carb-CEP-1 antibodies

CEP-1/carb-CEP-1	SE		OR^a^ (95 % CI)
	None	Any	
Controls	959 (49.87)	964 (50.13)	Reference 1.0
–/–	468 (37.23)	789 (62.77)	1.69^b^ (1.46, 1.97)
+/–	70 (13.65)	443 (86.35)	6.58^b^ (5.01, 8.65)
–/+	20 (32.79)	41 (67.21)	2.07^b^ (1.19, 3.61)
+/+	36 (8.91)	368 (91.09)	10.42^b^ (7.28, 14.90)

*SE* shared epitope, *CEP-1* citrullinated α-enolase peptide-1, *carb-CEP-1* carbamylated CEP-1, *CI* confidence interval, *RA* rheumatoid arthritis. ^a^Odds ratios (*OR*) were adjusted for age, gender and residential area. ^b^Statistically significant ORs

### Anti-carb-CEP-1 reactivity in relation to *PTPN22* in EIRA

We also investigated the association of *PTPN22* polymorphism with the presence of anti-carb-CEP-1 antibodies, in relation to the anti-CEP-1 antibody response. *PTPN22* differed from smoking and SE, in the sense that there was no difference in the association between CEP-1 single-positive (OR = 1.90; 95 % CI 1.54, 2.35) and carb-CEP-1 single-positive (OR = 2.05; 95 % CI 1.19, 3.51) disease (1.90 vs*.* 2.05, *p* = 0.85), and having both antibody reactivities (OR = 2.07; 95 % CI 1.64, 2.6) did not significantly alter the association either (1.90 vs*.* 2.07, *p* = 0.73) (Additional file 1: Table S2).

### Anti-carb-CEP-1 reactivity in relation to the overall ACPA response in EIRA

**Fig. 4:**
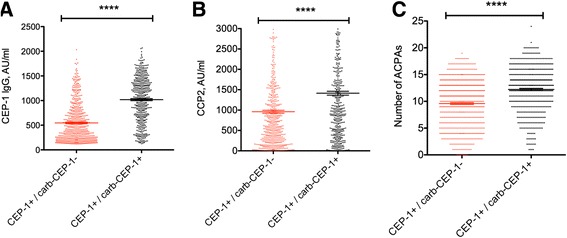
The anti-citrullinated protein antibody (ACPA) response is stronger in CEP-1/homocitrullinated CEP-1 peptide (*carb-CEP-1*) double-positive rheumatoid arthritis (RA) than in CEP-1 single-positive RA. Anti-CEP-1 IgG (**a**), anti-CCP2 IgG (**b**), and the number of ACPA fine specificities (**c**) were compared between the Swedish Epidemiological Investigation of Rheumatoid Arthritis (EIRA) cohort RA CEP-1^+^/carb-CEP-1^-^ and CEP-1^+^/carb-CEP-1^+^ subsets (****p < 0.0001). Data in **a** and **c** were generated using the immuno solid-phase allergen chip multiplex assay (ISAC) microarray; data in **b** were generated by ELISA. *AU* arbitrary units

Finally we analysed anti-CEP-1 IgG levels, anti-CCP2 IgG levels, and the number of ACPA fine specificities in CEP-1 single-positive RA, compared to CEP-1/carb-CEP-1 double-positive RA. Higher anti-CEP-1 and anti-CCP2 IgG levels were detected in the double-positive subset, and a higher number of ACPA fine specificities were recorded in CEP-1/carb-CEP-1 double-positive patients with RA, compared to CEP-1 single-positive patients; all values were significant with *p* values <0.0001 (Fig. 4). These results clearly demonstrate a stronger anti-CEP-1 antibody response and a stronger overall ACPA response in the subset with carb-CEP-1 (cross)-reactive antibodies.

## Discussion

This is the first report of an antibody response to carbamylated α-enolase in RA. Previous reports on anti-CarP antibodies in RA have focused mainly on carbamylated fibrinogen or the complex protein mixture of carbamylated fetal calf serum [13, 20–22, 24, 25], and recently a report was published on antibodies against carbamylated vimentin (26). Our study suggests that the anti-CarP antibody response in RA can be explained by cross-reactive ACPA. This conclusion was particularly evident from immunoblotting experiments using affinity-purified anti-CCP2 IgG molecules. Purified ACPA bound not only citrullinated α-enolase and citrullinated fibrinogen, but also carbamylated α-enolase and -fibrinogen, while unmodified proteins were not targeted, and importantly, ACPA-depleted IgG was not able to recognize citrullinated or carbamylated epitopes. Notably, the two earliest reports linking carbamylation to the development of arthritis in mouse models already describe cross-reactivity between citrulline- and homocitrulline-containing epitopes [17, 18].

Using a synthetic and artificial peptide based on the well-characterised CEP-1 epitope from citrullinated α-enolase [27, 28, 32] but with homocitrulline in place of citrulline (amino acids 9 and 15), we showed that approximately 20 % of patients with RA in the EIRA cohort had antibodies to carb-CEP-1. While the carb-CEP-1 peptide most likely does not represent an in vivo antigenic target (amino acid 9 and 15 of unmodified α-enolase are arginines, not lysines), and any biological and mechanistic implications based on the peptide data therefore are limited, the observed cross-reactivity between anti-CEP-1 IgG and carb-CEP-1 suggests that antibodies to citrullinated α-enolase can also bind to homocitrulline-containing epitopes. The fact that the anti-carb-CEP-1 antibody-positive subset of patients was almost exclusively confined to the CEP-1-positive population, together with the observation that CEP-1 could block carb-CEP-1 reactivity much more efficiently than carb-CEP-1 could block CEP-1 reactivity, clearly supports our interpretation; that is, that the reactivity measured as anti-CarP-antibodies are for the most part represented by cross-reactive ACPA.

The group of patients with anti-carb-CEP-1 antibodies had higher anti-CEP-1 antibody levels, but also higher anti-CCP2 antibody levels, and a broader ACPA repertoire (than anti-CEP-1 antibody-positive patients without anti-carb-CEP-1 antibodies), suggesting a stronger ACPA response in general in this group of patients, with epitope spreading and more promiscuous antigen-recognition, i.e., also including epitopes containing homocitrulline, which is structurally very similar to citrulline. Our gene-environment association data suggest that this extended antibody reactivity is influenced by *HLA-DRB1* SE alleles, but not by *PTPN22* or smoking.

Recently, anti-CarP antibodies have also been described in small subsets of patients with non-RA early arthritis [38], and in a large portion of patients with primary Sjögren’s syndrome [39], where the presence of anti-CarP antibodies correlated with disease severity. Contrary to our conclusion, these reports seem to indicate that this class of autoantibodies could be more of a general marker for inflammation than cross-reactive ACPAs, which would be specific for RA.

Taken together, our data seem to suggest that cross-reactivity between ACPA and anti-CarP antibodies in RA is a common phenomenon. Here, we have described this cross-reactivity in the context of the RA candidate autoantigen α-enolase. However, supported by recent work from Scinocca and colleagues, demonstrating cross-reactivity between citrullinated and carbamylated fibrinogen [20], we posit that this is also likely the case for other citrullinated/carbamylated antigens. In line with previous reports [20, 24], this cross-reactivity is not complete or consistent, and indeed a small percentage (3 %) of RA cases in our study demonstrated reactivity exclusively to the carb-CEP-1 peptide and not CEP-1. However, this subset of patients was almost completely eliminated when using a more stringent cut off for positivity, casting doubt on the existence of specific anti-CarP antibodies.

## Conclusions

ACPAs are cross-reactive with homocitrullinated epitopes on α-enolase. This calls into question the specificity of anti-carP antibodies, which we posit may be a cross-reactive subset of ACPAs.

## Additional file

Additional file 1: Supplementary tables and methods. **Table S1** α-enolase peptide sequences. **Table S2** Association between *PTPN22* polymorphism and RA in subgroups of patients, divided based on the presence/absence of anti-CEP-1 and anti-carb-CEP-1 IgG. (DOCX 22 kb)

## Abbreviations

ACPA: anti-citrullinated protein antibody
anti-CarP: anti-carbamylated protein antibody
carb-CEP-1: homocitrullinated CEP-1 peptide
CCP2: anti-cyclic citrullinated peptide, second generation
CEP-1: the immunodominant peptide epitope of citrullinated alpha-enolase
EIRA: Swedish Epidemiological Investigation of Rheumatoid Arthritis cohort
ELISA: enzyme-linked immunosorbent assay
FT: flow-through
HLA-DRB1: HLA class II histocompatibility antigen, DRB1-9 beta chain
IgG: immunoglobulin G
ISAC: immuno solid-phase allergen chip multiplex assay
OD: optical density
PAD: peptidylarginine deiminase
PTPN22: protein tyrosine phosphatase, non-receptor type 22 (lymphoid)
RA: rheumatoid arthritis
RT: room temperature
SE: shared epitope risk alleles
