# Supplementary material for: Antibodies to carbamylated α-enolase epitopes in rheumatoid arthritis also bind citrullinated epitopes and are largely indistinct from anti-citrullinated protein antibodies
Source: Arthritis Res Ther. 2016 May 4;18:96. doi: 10.1186/s13075-016-1001-6 (PMC4855497; doi:10.1186/s13075-016-1001-6)
Supplement: Additional file 1: — Supplementary tables and methods. Table S1 α-enolase peptide sequences. Table S2 Association between PTPN22 polymorphism and RA in subgroups of patients, divided based on the presence/absence of anti-CEP-1 and anti-carb-CEP-1 IgG. (DOCX 22 kb) [file 13075_2016_1001_MOESM1_ESM.docx]

**Additional file 1**

**Antibodies to carbamylated α-enolase epitopes in rheumatoid arthritis also bind citrullinated epitopes and are largely indistinct from anti-citrullinated protein antibodies**

Reed *et al.*

**SUPPLEMENTARY METHODS**

***Mass spectrometry***

Unmodified, citrullinated or carbamylated proteins (10 µg) were reduced, alkylated and digested in-solution according to Ytterberg et al^1^. After zip tipping (Merck Millipore Ltd, Tullagreen, Ireland), an amount corresponding to 2 pmol prior to precipitation and digestion were separated using on-line nLC-MS/MS (RP C18 column) and analyzed on a Q Exactive (Thermo Fisher Scientific, Bremen, Germany). The following gradient was used for the separation: 5-30% B in 35 min and 30-95% B in 5 min (A: 0.2 % formic acid in water; B: 0.2 % formic acid in acetonitrile), all at a flow rate of 300 nl/min. Mass lists were extracted using Raw2MGF v2.1.3^2^ and used to search a concatenated version of the SwissProt database (2013/4) using the Mascot search engine v2.3.02 ([www.matrixscience](http://www.matrixscience/), Matrix Science Ltd., London, UK). The following parameters were used: tryptic digestion (max 2 miscleavages); carbamidomethylation (C) as fixed modification; oxidation (M), pyroglutamate (Q), deamidation (N/Q), carbamylation (K) and citrullination (R) as variable modifications; 10 ppm as precursor tolerance; 0.1 Da as fragment tolerance. Peptides identified as carbamylated and citrullinated were validated manually.

**ISAC**

Serum samples from the EIRA cohort (n=2,836) and controls (n=373) were analysed for ACPA IgG fine specificities using the ImmunoCAP ISAC micro-array platform (Phadia Multiplexing Diagnostics GmbH, Vienna, Austria) as described previously^3^. In addition to CEP-1, carb-CEP-1 and REP-1, samples were analysed for reactivity to a custom array of 28 citrullinated peptides and their corresponding arginine-containing control peptides. Cutoffs for positivity (98^th^ percentile) for each antigen were calculated using differences in concentration between the RA and non-RA control samples. Fluorescence values were converted to Arbitrary Units (AU) using a standard curve derived from a poly-reactive serum sample.

**ELISA**

High-binding 96-well MaxiSorp ELISA plates (Thermo Scientific Nunc, Roskilde, Denmark) were coated with CEP-1, REP-1 and Carb-CEP-1 at 2µg/ml in carbonate buffer (50mM, pH 9.6) and incubated overnight at 4°C. After washing (PBS, 0.1% Tween) and blocking (PBS, 1% BSA) for 1 h at room temperature (RT), serum samples were added in duplicates diluted 1:100 in RIA buffer (1% BSA, 350 nM NaCl, 10 mM Tris-HCl, 1% Triton-X 100, 0.5% Na-deoxycholate, 0.1% SDS, pH 7.6), and incubated for 1h at RT. Serum samples were analysed in parallel for all three antigens on the same ELISA plate. Plates were washed before horse-radish peroxidase (HRP)-conjugated goat anti-human IgG (Jackson ImmunoResearch, West Grove PA, USA), diluted 1:10000 in RIA buffer, was added for 1h, RT. Following a final wash, TMB substrate (Sigma-Aldrich, St Louis MO, USA) was added and the colour-reaction stopped using 1M H_2_SO_4_ after approximately 15 minutes. Absorbance was determined at 450nm and presented as optical density (OD).

| Table S1 α-enolase peptide sequences | |
| --- | --- |
| Peptide | **Amino acid sequence** |
| CEP-1 | c-kiha-X-eifds-X-gnptve-c |
| carb-CEP-1 | c-kiha-H-eifds-H-gnptve-c |
| REP-1 | c-kiha-R-eifds-R-gnptve-c |

| **Table S2** Association between *PTPN22* polymorphism and RA in subgroups of patients, divided based on the presence/absence of anti-CEP-1 and anti-carb-CEP-1 IgG | | | |
| --- | --- | --- | --- |
| CEP-1/ carb-CEP-1 | *PTPN22* | | OR*(95% CI) |
|  | None | Any |  |
| controls | 1533 (79.18) | 403 (20.82) | ref. 1.0 |
| -/- | 1008 (73.15) | 370 (26.85) | **1.42 (1.20-1.67)** |
| +/- | 388 (66.78) | 193 (33.22) | **1.90 (1.54-2.35)** |
| -/+ | 44 (66.67) | 22 (33.33) | **2.05 (1.19-3.51)** |
| +/+ | 297 (65.71) | 155 (34.29) | **2.07 (1.64-2.60)** |

^*^Odds ratios (OR) were adjusted for age, gender and residential area. CEP-1 = citrullinated α-enolase peptide-1; carb-CEP-1 = carbamylated citrullinated α-enolase peptide-1; CI = confidence interval; RA = rheumatoid arthritis

**SUPPLEMENTARY REFERENCES**

1 Ytterberg AJ, Peltier J-B, van Wijk KJ. Protein profiling of plastoglobules in chloroplasts and chromoplasts. A surprising site for differential accumulation of metabolic enzymes. *Plant Physiol* 2006;**140**:984–97. doi:10.1104/pp.105.076083

2 Lyutvinskiy Y, Yang H, Rutishauser D, *et al.* In silico instrumental response correction improves precision of label-free proteomics and accuracy of proteomics-based predictive models. *Mol Cell Proteomics* 2013;**12**:2324–31. doi:10.1074/mcp.O112.023804

3 Hansson M, Mathsson L, Schlederer T, *et al.* Validation of a multiplex chip-based assay for the detection of autoantibodies against citrullinated peptides. *Arthritis Res Ther* 2012;**14**:R201. doi:10.1186/ar4039
